# Kidney health for all: caring for people, protecting the planet^*^

**DOI:** 10.1590/2175-8239-JBN-2026-0162en

**Published:** 2026-10-19

**Authors:** Raymond Vanholder, Dina Abdellatif, Augusto Cesar Soares dos Santos, Ricardo Correa-Rotter, Natarajan Gopalakrishnan, Bill Wang, Stefanos Roumeliotis, Alessandro Balducci, Ágnes Haris, Manjusha Yadla, Li-Li Hsiao

**Affiliations:** 1 Ghent University Hospital, Department of Internal Medicine and Pediatrics, Nephrology Section, Ghent, Belgium.; 2 European Kidney Health Alliance, Brussels, Belgium.; 3 Cairo University Hospital, Department of Nephrology, Cairo, Egypt.; 4 Faculdade Ciências Médicas de Minas Gerais, Belo Horizonte, MG, Brazil.; 5 Universidade Federal de Minas Gerais, Empresa Brasileira de Serviços Hospitalares, Hospital das Clínicas, Belo Horizonte, MG, Brazil.; 6 Instituto Nacional de Ciencias Médicas y Nutrición Salvador Zubirán, Mexico City, Mexico.; 7 Transplant Authority of Tamil Nadu, Chennai, India.; 8 Hong Kong Kidney Foundation, Hong Kong, China.; 9 Aristotle University of Thessaloniki, Medical School, AHEPA University Hospital, 2nd Department of Nephrology, Thessaloniki, Greece.; 10 Italian Kidney Foundation Italy, Rome, Italy.; 11 Péterfy Hospital, Department of Internal Medicine and Nephrology, Budapest, Hungary.; 12 Gandhi Medical College, Department of Nephrology, Hyderabad, Telangana, India.; 13 Brigham and Women’s Hospital, Harvard Medical School, Department of Medicine, Boston, United States.

**Keywords:** Environment, Green Nephrology, Prevention, Screening, Sustainable Kidney Care, World Kidney Day

## Abstract

The current kidney care model – focused on late-stage disease and in-center hemodialysis – is unsustainable due to costs, environmental burden, poor outcomes, and reduced quality of life. The 78th World Health Assembly’s recognition of kidney disease as a serious health threat presents a critical opportunity to reshape kidney care. In alignment with this, the 2026 World Kidney Day theme, “Kidney Health for All: Caring for People, Protecting the Planet” calls for a systematic change. A sustainable model must prioritize early detection and prevention, reducing the need for kidney replacement therapy. Transplantation and home dialysis benefit people with kidney failure, as well as the environment and society. Dialysis itself must become more eco-friendly without compromising quality of care, recognizing that planetary perturbations in turn affect kidney health. Conservative care should also be considered, particularly for elderly and frail patients, when the quality-of-life benefits outweigh those offered by dialysis. Achieving this shift requires coordinated action across all stakeholders; education and engagement of the public, policymakers, and health professionals to raise awareness about the threat of kidney disease; and an urgent move toward patient-centered care.

Chronic kidney disease (CKD) is one of the most common noncommunicable diseases globally^1,2^ and is linked to significant comorbidities, particularly cardiovascular disease, as well as premature mortality, societal costs, reduced productivity, and a considerable environmental footprint^3^. CKD disproportionately affects disadvantaged populations and minority groups^4^. Advanced CKD causes distressing symptoms, social isolation, and, in children, delays in growth and development.

Despite its wide-ranging impact, CKD remains underrecognized as a global health threat. Recently, CKD’s profile was raised by the World Health Organization Resolution on Kidney Health, approved at the 78th World Health Assembly^5^. The Resolution urges governments to integrate the prevention, early detection, and management of kidney disease into national noncommunicable disease strategies and to expand access to equitable, sustainable, high-quality kidney care, progressively including dialysis, transplantation, and conservative care within universal health coverage. In addition, World Kidney Day (WKD) was recognized as a key initiative for raising public awareness and promoting kidney health.

Sponsored and championed by Guatemala, the Resolution marked a pivotal step in establishing kidney disease as a global health priority. Guatemala’s leadership was instrumental in mobilizing broad support, particularly from countries most affected by the growing burden of CKD and inequities in access to care. The Resolution also recognized the strong influence of environmental factors on kidney health, highlighting the disproportionate impact of climate change on developing countries and small island States, as emphasized in the 77th WHA Resolution (see below).

These milestones provide the policy foundation for the 2026 WKD theme, “Kidney Health for All: Caring for People, Protecting the Planet”, which underscores the need to safeguard kidney health, address both the effects of the environment on kidney health and the environmental impact of kidney care, and shift health care models from a focus on late-stage treatment to early risk reduction and detection (Figure 1).

**Figure 1:**
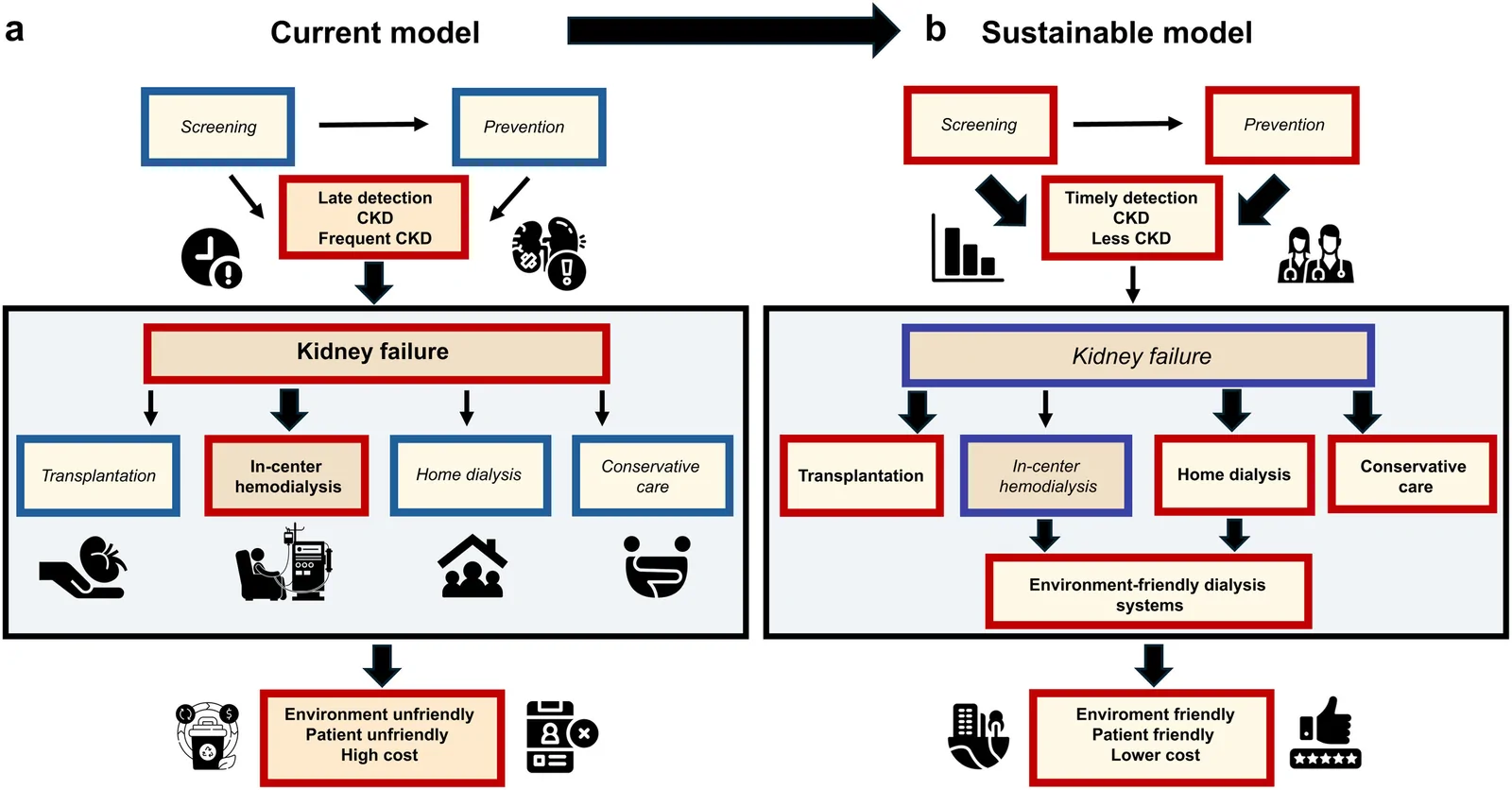
(a) Current model of kidney care. (b) Sustainable model of kidney care. Elements that are less or insufficiently prominent in a specific model are italicized with a blue frame; elements that are prominent in a specific model are bolded with a red frame. A yellow background indicates that an element is beneficial for sustainability, and an orange background indicates that it is disadvantageous. A light blue–shaded box with a black frame is used for kidney replacement therapy. The thickness of the arrows indicates the degree of impact on the subsequent element. CKD, chronic kidney disease; home dialysis, peritoneal dialysis and/or home hemodialysis.

This editorial calls for urgent, integrated action across early detection, prevention, equitable care, and environmental stewardship to advance both kidney and planetary health (Chart 1)^6^. It underscores the need for a more sustainable kidney care model with prevention and eco-friendliness as two main pillars (Figures 1 and 2)^6^, to which all other focal points are linked. We provide a general outline of measures that communities and governments can take, although it is virtually impossible to propose a ready-made plan that fits all settings. Kidney health plans need to be developed with the goal of a sustainable model, but they may require adaptation depending on local resources and circumstances.

**Chart 1:** Different actions to make kidney care sustainable, according to the topic and target.

Topic	Target population	Action
Prevention	People with diabetes	– Timely diagnosis – Adequate treatment
	People with hypertension	– Timely diagnosis – Adequate treatment
	Lifestyle risks jeopardizing kidney function	– Promote a healthy lifestyle and offer advice on correction (e.g., no smoking, reducing sedentarism, improving nutrition, and limiting alcohol consumption) – Limit exposure to nephrotoxins – Implement health–promoting taxation (e.g., salt, sugar, or fat taxes) – Conduct education campaigns
	Unfavorable living conditions; difficulties to reach quality care	– Improve living conditions (e.g., social measures to reduce insufficient building cooling, food insecurity, and poverty) – Address inequities (e.g., ethnic and gender disparities [gender refers to social or cultural rather than biological identity], health illiteracy, discrimination, and disadvantages in remote areas)
Early detection	People with risk factors for CKD General population aged >45 y	– Urine testing – Albuminuria testing – Screa/eGFR – Screening for CKD risk factors (mainly hypertension, diabetes, cardiovascular disease, and obesity) – Education of the public and caregivers – Ensuring that appropriate therapy reaches all eligible candidates
Environmental kidney threats	Global warming	– Urgently reduce GHG emissions – Adapt working conditions (adapt hydration and protection against pollution and nephrotoxins) – Adapt living conditions through building cooling and increased green spaces.
	Floods, hurricanes, and typhoons	– Urgently reduce GHG emission – Prevent floods – Avoid diseases propagated by floods (e.g., malaria and dengue) – Ensure safe water storage – Prevent water contamination in flood-prone areas.
	Fine particulate matter pollution	– Decrease and prevent the generation of fine particulate matter (industry, transport, and forest fires) – Minimize pollution and the release of particulate matter
Transplantation	Promotion of organ donation	– Provide clear guidelines on organ donation and transplantation – Act against donor and recipient exclusion based on questionable criteria – Encourage the use of all donor types (deceased, living, and after cardiac death) – Promote preemptive transplantation – Provide technical and institutional support for organizing transplantation programs in areas with poor transplantation rates – Establish guidelines on how to respond to paid donation – Develop education, press campaigns, and social media campaigns
	Promotion of organ transplantation	– Provide clear guidelines for organizing organ donation and transplantation programs – Provide technical and institutional support for organizing transplantation programs – Provide education – Conduct press and social media campaigns
	Declining graft function over time, post-transplantation complications	– Research and innovation to improve graft longevity (e.g., organ preservation, immunosuppression, and antifibrosis approaches)
Dialysis	Ecofriendly dialysisa	– Transparency about manufacturing and transport emissions – Spent dialysate and RO reject recycling – Decrease manufacturing and transport emissions – Register and diminish emissions at unit level – Recycling materials – Decrease water wastage – Waste triage – Peritoneal dialysis – Home hemodialysis
	Simpler and more compact dialysis systems	– Peritoneal dialysis – Research and innovation
Comprehensive conservative care	Preserve quality of life, particularly in frail and elderly patients	– Discuss the possibility of conservative care with patients – Engage in shared decision-making – Raise awareness among candidates for KRT and health professionals
Patient empowerment	All people with CKD	– Promote accurate and appropriate information delivery to all KRT candidates – Support shared decision-making – Facilitate patient–friendly units and hospitals – Increase nurse involvement in patient contacts – Organize kidney care discussion groups – Enhance patient involvement in research and registries – Provide patient training in communication skills
Kidney care in crises	All people with CKD All crisis situations All crisis-prone countries and regions	– Inclusion of kidney care in crisis preparedness plans – Raising awareness among authorities – Development of less resource-dependent therapies – Creation of independent disaster preparedness plans if kidney care is excluded from official plans
Advocacy	Entire community involved in kidney care	– Raising awareness about kidney health and the burden of kidney disease at all levels of society – Providing education and information

Abbreviations – CKD: chronic kidney disease; eGFR: estimated glomerular filtration rate; GHG: greenhouse gas; KRT: kidney replacement therapy; RO: reverse osmosis; Screa: serum creatinine.

Note – ^a^For more details, see Figure 2 and the study by Vanholder et al.^6^. The table summarizes several examples but is not exhaustive.

**Figure 2:**
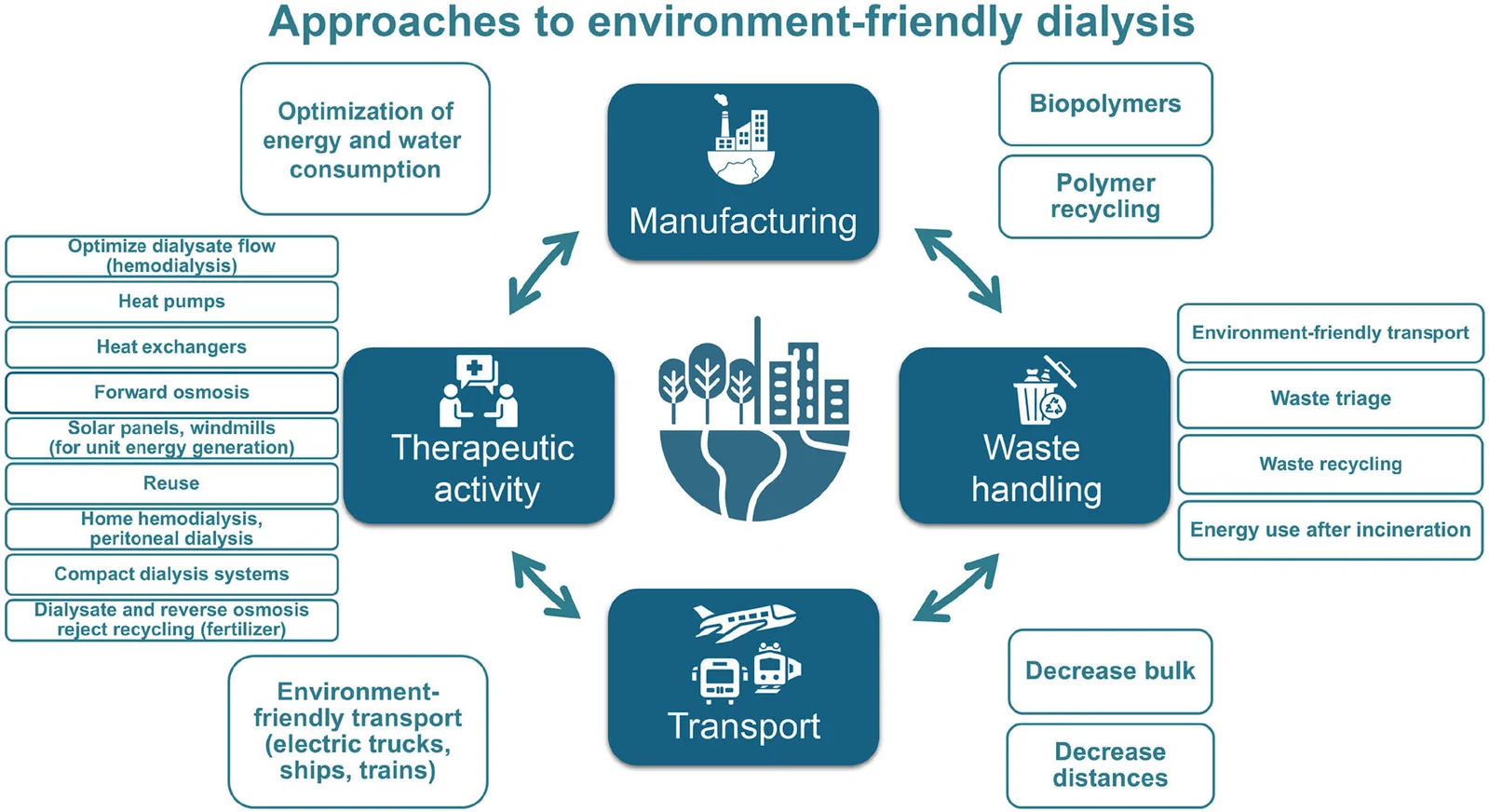
Approaches to environment-friendly dialysis, categorized by manufacturing, transport, therapeutic activity, and waste handling. The list is not exhaustive. For more detailed lists, see the study by Vanholder et al.^6^Environment friendliness should not be pursued at the expense of patient quality of life or clinical status. Compact dialysis systems use less water or recycle dialysate over sorbents.

## PREVENTION: THE FOUNDATION OF KIDNEY HEALTH

Beyond general primary and secondary preventive measures for all kidney conditions, prevention may require targeted therapies, including for rare kidney diseases^7^. Diabetes and hypertension – the leading causes of CKD – remain widely underdiagnosed and undertreated^8^. Kidney function is further compromised by modifiable lifestyle factors, including smoking, unhealthy nutritional habits (notably excess sugar and salt), alcohol use, obesity, exposure to nephrotoxins, physical inactivity, exposure to heat, environmental pollution, and agrochemicals. Addressing these could significantly reduce the CKD burden^9^. Prevention is beneficial across all country wealth levels but is especially critical where costly CKD therapies are inaccessible or inadequately reimbursed, resulting in premature death and catastrophic out-of-pocket health expenditures.

A shift toward prevention as the cornerstone of sustainable kidney care calls for simple yet effective public health strategies, such as promoting healthy diets or introducing health-oriented taxation (e.g., salt, sugar, or fat taxes)^9^. Currently, governments invest far more in cure than in prevention, and a more balanced allocation between the two is needed. Several governments are taking steps in that direction, sometimes in the context of a global cardiovascular health plan, but approaches differ depending on local conditions.

WKD promotes Eight Golden Rules for kidney health – simple, actionable lifestyle measures^10^: regular physical activity, a healthy diet, adequate hydration, blood glucose control, blood pressure management, avoiding smoking, using medications as prescribed, and regular kidney function testing. These principles provide a framework for kidney health promotion across populations and health care settings.

Because unfavorable living conditions and difficulties in reaching quality care have a negative impact on outcomes of noncommunicable diseases, including CKD^11^, prevention must also address social dimensions. These include food insecurity, poor food quality, poverty, remoteness (rural vs. urban), inadequate access to services, redlining, unfavorable living conditions (unhygienic and unsafe housing, inadequate building cooling, and polluted living areas), and lack of neighborhood greenery.

Public understanding of how the kidney functions and its threats is poor. Informative campaigns, like the European Renal Association’s ABCDE initiative, which highlights albuminuria, blood pressure, cholesterol, diabetes, and estimated glomerular filtration rate as key warning signs, should be widely disseminated^12^. The public should be encouraged to ask their primary care providers about these factors, as people currently do for their glucose and cholesterol.

However, educational outreach often misses those at highest risk of CKD: individuals with limited health literacy, language or cultural barriers, or lower socioeconomic status. Tailored approaches (videos, social media campaigns, and cartoons) are vital to reach these deprived populations regarding all aspects and levels of CKD.

## EARLY DETECTION: A CRITICAL, UNDERUTILIZED TOOL

CKD symptoms typically emerge at advanced stages, when therapies often can only delay, not prevent, kidney failure^13,14^. Serum creatinine remains the most widespread marker for routine assessment and monitoring of CKD but lacks sensitivity for early detection^15^, although early detection is essential to timely and effective prevention of progression. Albuminuria stands out as a simple, low-cost, and reliable early indicator of kidney microvascular damage and CKD, as well as related conditions such as hypertension, diabetes, and dyslipidemia^16^. However, urinary screening assessments, including albuminuria checks, are rarely performed, even in well-developed health care systems^17^.

Albuminuria testing can therefore detect hidden cases across the entire cardio-kidney-metabolic spectrum^16^, serving as an alarm for conditions that, if untreated, lead to irreparable organ damage, including to the kidneys. The progression of all conditions thus detected can be therapeutically decelerated, with the potential to reduce the pressing individual and societal burden of CKD and kidney failure. Modeling suggests that population-wide, timely albuminuria screening and treatment could lower the lifetime incidence of dialysis or transplantation by 21.8% and of cardiovascular disease by 12%^18,19^. Implementing universal screening from age 45 years, or even from 35 years onward, has been suggested to enhance cost-effectiveness and health system resilience^18^. Programs like the US Kidney Disease Screening and Awareness Program help promote early screening and detection of CKD^20^.

In addition, kidney disease should be a central focus not only within nephrology but also in diabetes, hypertension, cardiovascular disease, and obesity, as it accelerates negative outcomes in these conditions^21,22^. Highlighting this threat requires continued advocacy from the nephrology community^23^, particularly as several novel therapies are now available to delay progression^24^. For screening to have impact, it must be paired with education of the public and of frontline providers, such as general practitioners, nurses, pharmacists, and, particularly in low-resource settings, community health workers. Detection of CKD and its risk factors should be followed by effective therapies, given their wide-ranging benefits, and these therapies should be made universally accessible and affordable.

Despite its growing burden, awareness and early detection of CKD remain limited in low-and middle-income countries. A 2024 pilot study in high-risk groups in India reported 60% prevalence but extremely low awareness (16.5% in rural areas; 1.4% in urban areas)^25^. In Brazil, serum creatinine and albuminuria testing remain below recommended levels, limiting early CKD detection and opportunities for timely intervention^26^. These gaps underscore the urgent need to integrate essential screening tests, such as albuminuria and serum creatinine, into routine primary care, especially in regions where people often present with advanced disease.

## ENVIRONMENTAL RISKS: AN EMERGING CHALLENGE FOR KIDNEY HEALTH

We are witnessing rapid environmental and climatologic changes with profound health consequences. The kidneys, which are central to maintaining volume homeostasis, are especially vulnerable to these shifts.

Global warming increases the risk of heat stress and dehydration, which are major contributors to acute kidney injury and kidney stone development^27,28^ and, ultimately, progression to CKD^29^. Outdoor workers exposed to extreme heat are particularly at risk, especially when hydration is inadequate or working conditions are insufficiently adapted, as in Mesoamerican nephropathy, a rapidly progressing form of CKD identified in agricultural workers in (sub)tropical climates^30^.

Climate change also intensifies extreme weather events, such as floods, hurricanes, and typhoons. These events, combined with rising temperatures, increase the spread of tropical diseases, such as malaria and dengue, as well as water-borne disorders, such as leptospirosis and infectious diarrhea^31^, all of which can cause acute kidney injury. Floodwaters may also become nephrotoxic when contaminated with industrial or natural pollutants. Additionally, fine particulate matter from industry, transport, and forest fires has been linked to the prevalence of CKD^32^.

Vulnerable populations bear the brunt of these risks, often living in inadequately protected environments with limited access to cooling, greenery, and safe working conditions.

In 2024, the 77th World Health Assembly adopted a landmark Resolution on Climate Change and Health, recognizing the environmental crisis as a major threat to human well-being and calling for climate-resilient, low-carbon health systems. The Resolution urged Member States to incorporate health into national climate strategies and endorsed mechanisms such as the Alliance for Transformative Action on Climate and Health to support implementation^33^.

Building on this momentum, the 78th Resolution specifically emphasized coordinated global action on environmental threats to overall and kidney health. Together, these Resolutions create a compelling dual mandate: to place kidney health at the intersection of noncommunicable disease control and environmental stewardship, and to guide Member States toward more integrated, equitable, and sustainable health responses.

Addressing environmental risks, however, must go hand in hand with reducing the ecological footprint of kidney care itself (see below).

## ECOFRIENDLY KIDNEY FAILURE CARE: TOWARD GREENER NEPROLOGY

The ideal approach to preventing the negative impact of interventions is to take measures that ensure they are no longer needed. Accordingly, preventing kidney disease supports planetary sustainability by delaying or avoiding dialysis and reducing the use of pharmaceuticals, the production of which also has an environmental footprint. However, if drugs delay the progression of CKD and the need for kidney replacement therapy (KRT), this may offset the carbon footprint of drug production. A real-data secondary analysis of the placebo-controlled CREDENCE study, which investigated the impact of canagliflozin on outcomes, found a 20% to 25% reduction in greenhouse gas emissions over 2.6 years of follow-up in patients with type 2 diabetes who were not yet on KRT^34,35^.

## TRANSPLANTATION: ADVANCING ACCESS AND SUSTAINABILITY

Among KRTs, transplantation offers the best outcomes – lower societal costs, improved survival, better quality of life, and significantly less environmental impact compared with dialysis^36,37^.

Yet access to kidney transplantation varies widely across and within countries, influenced by health care infrastructure, socioeconomic status, and geography (rural vs. urban)^4^. Donation practices also differ: some countries rely heavily on living donors, whereas others focus mainly on deceased donation. In many areas, donation after cardiac death or preemptive transplantation remains underused, despite favorable outcomes^38,39^. Furthermore, potential donors and recipients are frequently disqualified on the basis of arbitrary criteria or prejudices, excluding specific social groups, age categories, women, individuals with nonessential comorbidities, and borderline donors. Excluding borderline donors also reduces the donor pool, despite evidence that kidneys from borderline donors are safe, provided careful assessment, long-term follow-up, and treatment of risk factors^40^. In low-resource settings, kidney transplantation programs are often underdeveloped or absent, further deepening health inequities and increasing economic burden.

Reducing transplantation disparities requires clear, globally endorsed guidelines on program design, ethical and legal frameworks on how to respond to paid donation, and technical and institutional support from countries or units with advanced transplantation programs. Efforts should also include public education programs to expand the donor pool and address cultural, religious, and societal concerns.

## SUSTAINABLE DIALYSIS: REDUCING ENVIRONMENTAL IMPACT WHILE IMPROVING ACCESS

Most people on KRT receive dialysis despite the many disadvantages mentioned above^37,41^. In recent years, the environmental burden of dialysis has emerged as an added concern.

The health care sector significantly contributes to environmental degradation^42^. Dialysis is one of the main contributors because of its repetitive and prolonged water and energy consumption, greenhouse gas emissions, and plastic waste generation^6^. Although therapeutic activities are a direct part of this process, approximately 70% of health care-related greenhouse gas emissions stem from the supply chain, largely related to manufacturing, transport, and waste handling^6,43,44^. In addition, substantial volumes of spent dialysate and reverse osmosis reject are usually discarded through the drain system, a particular issue in arid regions or during droughts^6,45^.

Urgent action is needed: investment in ecofriendly dialysis technologies that prioritize patient safety by reducing toxicity from microplastics and eluates, while also improving the treatment experience by addressing stressors such as excessively noisy machines; critical review of clinical procedures to reduce the environmental footprint without affecting treatment quality; and greater transparency about the burdens of manufacturing and transport and how they are addressed (Figure 2)^6^. This responsibility requires collaboration among industry, physicians, patients, nurses, engineers, chemists, and environmental scientists.

Home-based therapies – peritoneal dialysis and most home hemodialysis regimens, except daily extended dialysis – offer environmental advantages compared with in-center hemodialysis, including reduced patient and personnel transportation needs, lower energy consumption for room temperature control, lower reverse osmosis plant electricity consumption, and, with compact hemodialysis systems and peritoneal dialysis, less water consumption. Peritoneal dialysis has a smaller footprint than in-center hemodialysis^43,46,47^, even though transporting its bulkier supplies may still generate substantial emissions, particularly when transport distance is high^46^. Most home hemodialysis regimens are also suggested to show a benefit compared with in-center hemodialysis, except for 6 × 8-hour extended hemodialysis (300 mL/min dialysate flow)^47^. Incremental dialysis may further decrease environmental burden, together with dialysate flow optimization^48^, if clinically suitable and provided informed consent is obtained.

Peritoneal dialysis and existing compact hemodialysis systems can also help expand dialysis access in low-resource settings and during crises. In addition, several systems that regenerate dialysate over sorbents are currently under development or undergoing preliminary clinical testing but are not yet available for widespread clinical use^49^. However, to achieve availability in emerging countries, barriers such as import taxes or transport costs must be addressed (e.g., through local manufacturing).

## COMPREHENSIVE CONSERVATIVE CARE: INDISPENSABLE BUT OFTEN FORGOTTEN

Comprehensive conservative or palliative care is the third option for treating kidney failure, alongside transplantation and dialysis. This approach focuses on maximizing quality of life through full medical support, without initiating KRT^50^.

Conservative care is particularly appropriate for frail and elderly people, many of whom experience a rapid decline in functional status and increased mortality in the first year after starting dialysis^51^. People with CKD stages 3 to 5 preferred conservative care if dialysis meant more hospital visits or restricted their ability to travel^52^. Despite this, conservative care often remains underutilized^50,53^.

Educational initiatives targeting both people with kidney disease and health care professionals should increase awareness and understanding of conservative care. Broadly promoting this approach can help ensure that people receive treatments aligned with their values, circumstances, and wishes, while reducing unnecessary medical, economic, and environmental burdens.

## PATIENT EMPOWERMENT: THE ELEPHANT IN THE ROOM

Many people report that the information provided before starting KRT is of average to poor quality, particularly in countries with lower gross domestic product^54^.

Paternalistic decision-making remains common, overlooking opportunities for early modality planning, vascular access creation, and elective KRT initiation. By contrast, shared decision-making actively engages patients in treatment choices, enhancing satisfaction, quality of life, outcomes, transplantation, and home dialysis rates, and reducing reliance on in-center hemodialysis^54,55^.

Current hospital structures remain highly patient-unfriendly, so even vocal people are poorly heard. Readily accessible, smaller, hospital-independent outpatient units, greater nurse involvement in consultations, and discussion groups with active patient participation are possible innovative options, alongside several educational and informative initiatives elaborated throughout this text. Support from patient organizations could help advance these developments.

Effective predialysis care, accurate risk prediction, and strategic modality planning are crucial for improving outcomes and reducing health care costs, but could be optimized, particularly in lower-resource areas, by preventing late referral, fragmented care, inadequate patient education, poor adherence, and limited communication.

Patient involvement is also crucial in research design, data interpretation, and registry development, helping to steer efforts in directions that truly matter to them. Patients should also be encouraged to participate in training programs that build confidence and communication skills, empowering them to become active care partners and shape the future of kidney health^56^.

## KIDNEY CARE IN CRISIS SITUATIONS: ADAPTING TO A CHANGING WORLD

The number of people affected by crisis situations is steadily increasing, driven by population growth, climate change, and growing geopolitical instability. These crises range from natural disasters (earthquakes, floods, extreme weather, and pandemics) to human-made emergencies, particularly armed conflicts^57^.

People with kidney diseases are particularly vulnerable as they depend on specialized medication and resource-intensive treatments such as dialysis. These treatments require skilled staff, clean water, electricity, functioning machines, transportation to supply stock, and reliable infrastructure – resources typically compromised in crises^57^.

Missed dialysis sessions can rapidly lead to life-threatening complications, such as hyperkalemia, which may require urgent evacuation^58^. Interruption of drug therapies may accelerate CKD progression or cause kidney graft rejection. Despite these risks, people with kidney diseases are rarely considered in official disaster plans.

As crises become more frequent and severe, kidney health should be integrated into disaster planning and emergency protocols. Authorities should be made aware of the nephrology community’s specific needs through strong, coordinated advocacy. If kidney care is not included in general disaster plans, nephrology communities and nephrologists should develop their own. At the same time, there is an urgent need for treatment strategies that are less dependent on resources.

## CALL TO ACTION: A COLLECTIVE AND GLOBAL RESPONSIBILITY

The traditional kidney care model centered on in-center hemodialysis is no longer sustainable – ecologically, economically, or ethically (Figure 1). People living with CKD urgently need better quality of life and care. Transformation requires a holistic approach (Chart 1), with all stakeholders considering each of the key areas discussed above and adapting them to local situations, infrastructure, and resources.

WKD 2026 amplifies this need, issuing a call to global action:

- The public should be educated to ask care providers about their kidney parameters and function.
- Workers, particularly those employed outdoors in agriculture and construction, should be sensitized to occupational threats from heat and environmental pollution and to strategies for mitigating these risks to preserve kidney health.
- People with kidney disease and their communities should be empowered to take an active role in shaping care pathways that meet their needs and serve their interests.
- Health care professionals must shift emphasis toward integrated, preventive, community-based care that enhances well-being and environmental responsibility, with a focus on early disease and out-of-hospital approaches.
- Researchers and industry must challenge the status quo and prioritize innovation, striving for equity, flexibility, patient friendliness, and sustainability.
- Policy makers and administrators need to recognize the cardio-kidney-metabolic cluster as a critical public health threat, prompting system redesign and promoting sustainable kidney care through funding and reimbursement initiatives.
- Patient advocacy organizations must be recognized as essential partners in the design, implementation, and evaluation of policy initiatives to ensure the system is built around patient-specific needs. However, active advocacy is a responsibility of the entire kidney care community.
- Global health partnerships should connect these efforts, advocating at all levels for cohesive, future-ready kidney care and global health.

Widespread lack of awareness – among the public, policy makers, and even health professionals – contributes to persistent inequities across socioeconomic, gender (social or cultural rather than biological identity), ethnic, national, and regional lines. Tackling this requires robust education that highlights the public health threats and overall burden imposed by kidney damage and kidney diseases.

Given demographic and geopolitical trends, appropriate, well-planned screening, early detection, and prevention are the first and foremost steps toward achieving sustainability goals. These measures reduce complications, progression to advanced kidney disease, and the need for KRT. Prevention will ultimately also favor global health by mitigating the course of diseases that frequently cause or complicate CKD and/or are accelerated by CKD, significantly decreasing personal and societal problems that arise even in robust social security systems. In the absence of universal coverage, this will have even greater benefits, by avoiding early fatal outcomes and devastating financial consequences for many.

## CONCLUSION: ALIGNING KIDNEY HEALTH WITH GLOBAL HEALTH AND SUSTAINABILITY AGENDAS

The recognition of kidney diseases as a global priority by the World Health Organization at the 78th World Health Assembly marks a pivotal moment^5^. It provides a comprehensive mandate for urgent action and a strong foundation for national and international advocacy.

The 2026 WKD theme catalyzes the integration of kidney health and care into broader health and sustainability agendas. This shift to patient-centered models must be championed by all stakeholders who share responsibility for realizing this approach across health, environment, labor, and policy sectors, through aligned partnerships, movements, and coalitions with groups that have parallel interests across organ specialties.

This publication and WKD 2026 issue an urgent, unified call to prioritize kidney health as a pillar of a healthier, fairer, and more sustainable future. The decisions made now will determine the lives of millions for decades to come.

## Data availability

No new data were generated or analyzed in this study.
