# Supplementary material for: Kidney health for all: caring for people, protecting the planet
Source: J Bras Nefrol. 2026 Oct 19;48(4):e20260162. doi: 10.1590/2175-8239-JBN-2026-0162en (PMC13682596; doi:10.1590/2175-8239-JBN-2026-0162en)
Supplement: Translation

# Translation: Saúde renal para todos: cuidar de pessoas e proteger o planeta^*^

**DOI:** 10.1590/2175-8239-JBN-2026-0162pt

**Authors:** Raymond Vanholder, Dina Abdellatif, Augusto Cesar Soares dos Santos, Ricardo Correa-Rotter, Natarajan Gopalakrishnan, Bill Wang, Stefanos Roumeliotis, Alessandro Balducci, Ágnes Haris, Manjusha Yadla, Li-Li Hsiao

**Affiliations:** 1 Ghent University Hospital, Department of Internal Medicine and Pediatrics, Nephrology Section, Ghent, Belgium.; 2 European Kidney Health Alliance, Brussels, Belgium.; 3 Cairo University Hospital, Department of Nephrology, Cairo, Egypt.; 4 Faculdade Ciências Médicas de Minas Gerais, Belo Horizonte, MG, Brasil.; 5 Universidade Federal de Minas Gerais, Empresa Brasileira de Serviços Hospitalares, Hospital das Clínicas, Belo Horizonte, MG, Brasil.; 6 Instituto Nacional de Ciencias Médicas y Nutrición Salvador Zubirán, Mexico City, Mexico.; 7 Transplant Authority of Tamil Nadu, Chennai, India.; 8 Hong Kong Kidney Foundation, Hong Kong, China.; 9 Aristotle University of Thessaloniki, Medical School, AHEPA University Hospital, 2nd Department of Nephrology, Thessaloniki, Greece.; 10 Italian Kidney Foundation Italy, Rome, Italy.; 11 Péterfy Hospital, Department of Internal Medicine and Nephrology, Budapest, Hungary.; 12 Gandhi Medical College, Department of Nephrology, Hyderabad, Telangana, India.; 13 Brigham and Women’s Hospital, Harvard Medical School, Department of Medicine, Boston, United States.

**Keywords:** Meio Ambiente, Nefrologia Verde, Prevenção, Rastreamento, Cuidados Renais Sustentáveis, Dia Mundial do Rim

## Abstract

O atual modelo de cuidados renais — centrado na doença em estágio avançado e na hemodiálise em centro especializado — é insustentável devido aos custos, ao impacto ambiental, aos desfechos desfavoráveis e à redução da qualidade de vida. O reconhecimento da doença renal como uma grave ameaça à saúde pela 78ª Assembleia Mundial da Saúde oferece uma oportunidade crucial para reformular o tratamento renal. Em consonância com esse reconhecimento, o tema do Dia Mundial do Rim de 2026, “Saúde Renal para Todos: Cuidar de Pessoas e Proteger o Planeta”, clama por uma mudança sistêmica. Um modelo sustentável deve priorizar a detecção precoce e a prevenção, reduzindo a necessidade de terapia renal substitutiva. O transplante e a diálise domiciliar trazem benefícios às pessoas com insuficiência renal, bem como ao meio ambiente e à sociedade. A própria diálise deve se tornar mais sustentável do ponto de vista ambiental, sem comprometer a qualidade do atendimento, reconhecendo que as mudanças ambientais globais, por sua vez, afetam a saúde renal. O tratamento conservador também deve ser considerado, especialmente para pacientes idosos e frágeis, quando os benefícios à qualidade de vida superarem os oferecidos pela diálise. A concretização dessa mudança requer uma ação coordenada entre todas as partes interessadas; a educação e o engajamento do público, dos formuladores de políticas e dos profissionais de saúde para aumentar a conscientização sobre a ameaça que representam as doenças renais; e uma transição urgente para um cuidado centrado no paciente.

A doença renal crônica (DRC) é uma das doenças não transmissíveis mais comuns em todo o mundo^1,2^ e está associada a comorbidades significativas, particularmente doenças cardiovasculares, bem como à mortalidade prematura, custos sociais, redução da produtividade e uma pegada ambiental considerável^3^. A doença afeta de forma desproporcional populações desfavorecidas e grupos minoritários^4^. A DRC em estágio avançado causa sintomas debilitantes, isolamento social e, em crianças, atrasos no crescimento e no desenvolvimento.

Apesar de seu amplo impacto, a DRC continua sendo subestimada como uma ameaça à saúde global. Recentemente, a visibilidade da DRC foi ampliada pela Resolução da Organização Mundial da Saúde sobre Saúde Renal, aprovada na 78ª Assembleia Mundial da Saúde^5^. A Resolução incita os governos a integrarem prevenção, detecção precoce e manejo da doença renal às estratégias nacionais para doenças não transmissíveis e a ampliarem o acesso a cuidados renais equitativos, sustentáveis e de alta qualidade, incluindo progressivamente a diálise, o transplante e o tratamento conservador na cobertura universal de saúde. Além disso, o Dia Mundial do Rim (DMR) foi reconhecido como uma iniciativa fundamental para aumentar a conscientização pública e promover a saúde renal.

Patrocinada e promovida pela Guatemala, a Resolução marcou um passo decisivo para estabelecer a doença renal como uma prioridade de saúde global. A liderança da Guatemala foi fundamental para mobilizar amplo apoio, especialmente por parte dos países mais afetados pelo crescente ônus da DRC e pelas desigualdades no acesso aos cuidados de saúde. A Resolução também reconheceu a forte influência dos fatores ambientais na saúde renal, destacando o impacto desproporcional das mudanças climáticas nos países em desenvolvimento e nos pequenos Estados insulares, conforme enfatizado na Resolução da 77ª Assembleia Mundial da Saúde (ver abaixo).

Esses marcos fornecem a base de políticas para o tema do DMR 2026, “Saúde Renal para Todos: Cuidar de Pessoas e Proteger o Planeta”, que ressalta a necessidade de preservar a saúde renal, abordar tanto os efeitos do meio ambiente sobre a saúde renal quanto o impacto ambiental dos cuidados renais e transitar de modelos de assistência à saúde centrados no tratamento em estágios avançados para redução precoce de riscos e detecção precoce (Figura 1).

**Figura 1:** (a) Modelo atual de cuidados renais. (b) Modelo sustentável de cuidados renais. Os elementos menos proeminentes ou insuficientemente destacados em um modelo específico estão em itálico e realçados por uma moldura azul; os elementos proeminentes em um modelo específico estão em negrito e realçados por uma moldura vermelha. O fundo amarelo indica que um elemento é benéfico para a sustentabilidade, e o fundo laranja indica que é desfavorável. Uma caixa sombreada em azul claro com moldura preta é utilizada para indicação de terapia renal substitutiva. A espessura das setas indica o grau de impacto sobre o elemento subsequente. DRC, doença renal crônica; diálise domiciliar, diálise peritoneal e/ou hemodiálise domiciliar.

Este editorial preconiza uma ação urgente e integrada, abrangendo a detecção precoce, a prevenção, o cuidado equitativo e a gestão ambiental responsável, com o objetivo de promover tanto a saúde renal quanto a saúde do planeta (Quadro 1)^6^. Ressalta a necessidade de um modelo de cuidados renais mais sustentável, tendo a prevenção e a sustentabilidade ambiental como dois pilares principais (Figuras 1 e 2)^6^, aos quais todos os demais pontos centrais estão relacionados. Apresentamos uma visão geral das medidas que as comunidades e os governos podem adotar, embora seja praticamente impossível propor um plano preestabelecido que se adapte a todos os contextos. Os planos de saúde renal devem ser desenvolvidos com o objetivo de alcançar um modelo sustentável; porém, podem demandar adaptações, dependendo dos recursos e das circunstâncias locais.

**Quadro 1:** Diferentes ações para tornar o cuidado renal sustentável, de acordo com o tema e o objetivo.

Tema	População-alvo	Ação
Prevenção	Pessoas com diabetes	– Diagnóstico oportuno – Tratamento adequado
	Pessoas com hipertensão	– Diagnóstico oportuno – Tratamento adequado
	Riscos relacionados ao estilo de vida que comprometem a função renal	– Promover um estilo de vida saudável e prestar aconselhamento para a correção de hábitos inadequados (por exemplo, não fumar, reduzir o sedentarismo, melhorar a alimentação e limitar o consumo de álcool) – Limitar a exposição a nefrotoxinas – Implementar tributação destinada à promoção da saúde (por exemplo, impostos sobre sal, açúcar ou gordura) – Realizar campanhas educativas
	Condições de vida desfavoráveis; dificuldades no acesso a cuidados de qualidade	– Melhorar as condições de vida (p. ex., medidas sociais para reduzir a climatização insuficiente dos ambientes, a insegurança alimentar e a pobreza) – Abordar as desigualdades (p. ex., disparidades étnicas e de gênero [gênero refere-se à identidade social ou cultural, e não à identidade biológica], baixo letramento em saúde, discriminação e desvantagens em áreas remotas)
Detecção precoce	Pessoas com fatores de risco para DRC População geral com idade >45 anos	– Exames de urina – Exames de albuminúria – CrS/TFGe – Rastreamento de fatores de risco para DRC (principalmente hipertensão, diabetes, doenças cardiovasculares e obesidade) –Educação da população e dos cuidadores –Garantir que o tratamento adequado chegue a todos os candidatos elegíveis
Ameaças ambientais à saúde renal	Aquecimento global	– Reduzir urgentemente as emissões de GEE – Adaptar as condições de trabalho (adaptar hidratação e proteção contra poluição e nefrotoxinas) – Adaptar as condições de vida por meio da climatização dos ambientes e da ampliação de áreas verdes
	Enchentes, furacões e tufões	– Reduzir urgentemente as emissões de GEE – Prevenir inundações – Prevenir doenças transmitidas por inundações (p. ex., malária e dengue) – Garantir o armazenamento seguro de água – Prevenir a contaminação da água em áreas propensas a inundações
	Poluição por material particulado fino	– Reduzir e prevenir a geração de material particulado fino (indústria, transportes e incêndios florestais) – Minimizar a poluição e a emissão de material particulado
Transplante	Promoção da doação de órgãos	– Fornecer diretrizes claras sobre doação e transplante de órgãos – Atuar contra a exclusão de doadores e receptores com base em critérios questionáveis – Incentivar o uso de todos os tipos de doadores (falecidos, vivos e após morte cardíaca) – Promover o transplante preemptivo – Oferecer suporte técnico e institucional para a organização de programas de transplante em regiões com baixas taxas desses procedimentos –Estabelecer diretrizes sobre como lidar com a doação remunerada –Desenvolver campanhas educacionais, na imprensa e nas redes sociais
	Promoção do transplante de órgãos	– Fornecer diretrizes claras para a organização de programas de doação e transplante de órgãos – Oferecer suporte técnico e institucional para a organização de programas de transplante – Promover ações educativas – Realizar campanhas na imprensa e nas redes sociais
	Declínio da função do enxerto ao longo do tempo, complicações pós-transplante	– Pesquisa e inovação para aumentar a longevidade do enxerto (p. ex., preservação de órgãos, imunossupressão e terapias antifibróticas)
Diálise	Diálise sustentávela	– Transparência quanto às emissões decorrentes da fabricação e do transporte – Reciclagem do dialisato utilizado e do rejeito da OR – Redução das emissões decorrentes da fabricação e do transporte – Registro e redução das emissões em nível de unidade – Reciclagem de materiais – Redução do desperdício de água – Triagem de resíduos – Diálise peritoneal – Hemodiálise domiciliar
	Sistemas de diálise mais simples e compactos	– Diálise peritoneal – Pesquisa e inovação
Tratamento conservador abrangente	Preservar a qualidade de vida, especialmente em pacientes frágeis e idosos	– Discutir a possibilidade de tratamento conservador com os pacientes – Incentivar a tomada de decisão compartilhada – Promover a conscientização de candidatos à TRS e profissionais de saúde
Empoderamento do paciente	Todas as pessoas com DRC	–Promover a divulgação de informações precisas e adequadas a todos os candidatos à TRS – Apoiar a tomada de decisão compartilhada –Promover unidades e hospitais que priorizem o bem-estar do paciente –Aumentar o envolvimento dos profissionais de enfermagem no contato com os pacientes –Organizar grupos de discussão sobre cuidados renais –Fortalecer o envolvimento dos pacientes em pesquisas e registros –Oferecer treinamento aos pacientes em habilidades de comunicação
Cuidados renais em situações de crise	Todas as pessoas com DRC Todas as situações de crise Todos os países e regiões propensos a crises	– Inclusão dos cuidados renais nos planos de preparação para situações de crise – Conscientização das autoridades – Desenvolvimento de terapias menos dependentes de recursos – Elaboração de planos de contingência independentes, caso os cuidados renais sejam excluídos dos planos oficiais
Conscientização e mobilização	Toda a comunidade envolvida nos cuidados renais	– Conscientização sobre a saúde renal e o ônus das doenças renais em todos os níveis da sociedade – Fornecer educação e informação

Abreviaturas – DRC: doença renal crônica; TFGe: taxa de filtração glomerular estimada; GEE: gases de efeito estufa; TRS: terapia renal substitutiva; OR: osmose reversa; CrS: creatinina sérica.

Note – ^a^Para mais detalhes, consulte a Figura 2 e o estudo de Vanholder et al.^6^. A tabela resume diversos exemplos, mas não contempla todas as possibilidades.

**Figura 2:** Abordagens para uma diálise sustentável, categorizadas por fabricação, transporte, atividade terapêutica e manejo de resíduos. A lista não contempla todas as possibilidades. Para listas mais detalhadas, consulte o estudo de Vanholder et al.^6^ A sustentabilidade ambiental não deve ser buscada em detrimento da qualidade de vida ou do quadro clínico do paciente. Os sistemas compactos de diálise utilizam menos água ou reciclam o dialisato por meio de sorventes.

## PREVENÇÃO: A BASE DA SAÚDE RENAL

Além das medidas gerais de prevenção primária e secundária para todas as doenças renais, a prevenção pode exigir terapias direcionadas, inclusive para doenças renais raras^7^. Diabetes e hipertensão — as principais causas da DRC — permanecem amplamente subdiagnosticados e subtratados^8^. A função renal é ainda mais comprometida por fatores de estilo de vida modificáveis, incluindo tabagismo, hábitos alimentares inadequados (notadamente o consumo excessivo de açúcar e sal), consumo de álcool, obesidade, exposição a nefrotoxinas, sedentarismo, exposição ao calor, poluição ambiental e agrotóxicos. Abordar esses fatores poderia reduzir significativamente o ônus da DRC^9^. A prevenção é benéfica em países de todos os níveis de renda, mas é especialmente crítica em locais onde as terapias de alto custo para a DRC são inacessíveis ou reembolsadas de forma inadequada, resultando em morte prematura e despesas diretas em saúde com impacto catastrófico.

Uma mudança em direção à prevenção como pilar fundamental do cuidado renal sustentável requer estratégias de saúde pública simples, porém eficazes, como a promoção de dietas saudáveis ou a introdução de tributação voltada à saúde (por exemplo, impostos sobre sal, açúcar ou gordura)^9^. Atualmente, os governos investem muito mais no tratamento do que na prevenção, sendo necessária uma alocação mais equilibrada de recursos entre ambos. Diversos governos estão adotando medidas nesse sentido, por vezes no contexto de um plano global de saúde cardiovascular, mas as abordagens variam de acordo com as condições locais.

O DMR promove as Oito Regras de Ouro para a saúde renal – medidas simples e práticas relacionadas ao estilo de vida^10^: prática regular de atividade física, alimentação saudável, hidratação adequada, controle da glicemia, monitoramento da pressão arterial, evitar o tabagismo, uso de medicamentos conforme prescrição médica e avaliação regular da função renal. Esses princípios fornecem uma estrutura para a promoção da saúde renal em todas as populações e contextos de atenção à saúde.

Como as condições de vida desfavoráveis e as dificuldades no acesso a cuidados de qualidade têm um impacto negativo nos desfechos das doenças não transmissíveis, incluindo a DRC^11^, a prevenção deve também abordar as dimensões sociais. Estas incluem insegurança alimentar, má qualidade dos alimentos, pobreza, isolamento geográfico (áreas rurais vs. urbanas), acesso inadequado aos serviços, discriminação no acesso a serviços financeiros (*redlining*), condições de vida desfavoráveis (moradias insalubres e não seguras, climatização inadequada dos ambientes e áreas residenciais poluídas) e falta de áreas verdes nos bairros.

A compreensão da população sobre o funcionamento dos rins e os fatores que os ameaçam é bastante limitada. Campanhas informativas, como a iniciativa ABCDE da *European Renal Association*, que destaca a albuminúria, a pressão arterial, o colesterol, o diabetes e a taxa de filtração glomerular estimada como principais sinais de alerta, devem ser amplamente divulgadas^12^. A população deve ser incentivada a consultar seus profissionais de atenção primária sobre esses fatores, da mesma forma que se faz atualmente em relação à glicemia e ao colesterol.

No entanto, as ações educativas muitas vezes não alcançam aqueles com maior risco de DRC: indivíduos com alfabetização em saúde limitada, barreiras linguísticas ou culturais ou com nível socioeconômico mais baixo. Abordagens personalizadas (vídeos, campanhas nas redes sociais e ilustrações) são essenciais para abordar essas populações desfavorecidas, contemplando todos os aspectos e estágios da DRC.

## DETECÇÃO PRECOCE: UMA FERRAMENTA FUNDAMENTAL E SUBUTILIZADA

Os sintomas da DRC geralmente surgem em estágios avançados, quando as terapias muitas vezes podem apenas retardar, e não prevenir, a insuficiência renal^13,14^. A creatinina sérica continua sendo o marcador mais utilizado para a avaliação e o monitoramento de rotina da DRC, mas apresenta baixa sensibilidade para a detecção precoce^15^, embora essa detecção precoce seja essencial para a prevenção oportuna e eficaz da progressão da doença. A albuminúria destaca-se como um indicador precoce simples, de baixo custo e confiável de danos microvasculares renais e de DRC, bem como de condições relacionadas, como hipertensão, diabetes e dislipidemia^16^. No entanto, os exames de rastreamento urinário, incluindo a verificação da albuminúria, são raramente realizados, mesmo em sistemas de saúde bem desenvolvidos^17^.

O exame de albuminúria pode, portanto, detectar casos não diagnosticados em todo o espectro cardiovascular-renal-metabólico^16^, servindo como um alerta para condições que, se não tratadas, levam a danos irreversíveis aos órgãos, incluindo os rins. A progressão de todas as condições assim detectadas pode ser retardada por meio de intervenções terapêuticas, com o potencial de reduzir o pesado ônus individual e social da DRC e da insuficiência renal. Modelos sugerem que o rastreamento oportuno da albuminúria em toda a população, juntamente com o tratamento, poderia reduzir em 21,8% a incidência de diálise ou transplante ao longo da vida e em 12% a de doenças cardiovasculares^18,19^. Tem-se sugerido a implementação do rastreamento universal a partir dos 45 anos, ou mesmo a partir dos 35 anos, para aumentar a relação custo-efetividade e a resiliência do sistema de saúde^18^. Programas como o *Kidney Disease Screening and Awareness Program*, dos Estados Unidos, ajudam a promover o rastreamento e a detecção precoces da DRC^20^.

Além disso, a doença renal deve ser um tema central não apenas na nefrologia, mas também no contexto do diabetes, da hipertensão, das doenças cardiovasculares e da obesidade, uma vez que acelera os desfechos adversos nessas condições^21,22^. Destacar essa ameaça requer um trabalho contínuo de conscientização e mobilização por parte da comunidade nefrológica^23^, especialmente considerando que diversas novas terapias estão atualmente disponíveis para retardar a progressão da doença^24^. Para que o rastreamento tenha impacto, ele deve ser associado a ações de educação junto à população e aos profissionais de saúde que atuam na linha de frente, como clínicos gerais, enfermeiros, farmacêuticos e, especialmente em contextos com poucos recursos, agentes comunitários de saúde. A detecção da DRC e de seus fatores de risco deve ser seguida pelo uso de terapias eficazes, considerando seus amplos benefícios, e essas terapias devem tornar-se universalmente acessíveis e economicamente viáveis.

Apesar de seu ônus crescente, a conscientização e a detecção precoce da DRC permanecem limitadas em países de baixa e média renda. Um estudo-piloto realizado em 2024 com grupos de alto risco na Índia relatou uma prevalência de 60%, mas níveis extremamente baixos de conscientização (16,5% em áreas rurais; 1,4% em áreas urbanas)^25^. No Brasil, a realização de exames de creatinina sérica e albuminúria permanecem abaixo dos níveis recomendados, limitando a detecção precoce da DRC e as oportunidades de intervenção oportuna^26^. Essas lacunas ressaltam a necessidade urgente de integrar exames essenciais de rastreamento, como albuminúria e creatinina sérica, à rotina da atenção primária, especialmente em regiões onde os pacientes frequentemente se apresentam com doença em estágio avançado.

## RISCOS AMBIENTAIS: UM DESAFIO EMERGENTE PARA A SAÚDE RENAL

Estamos testemunhando rápidas mudanças ambientais e climáticas, com profundas consequências para a saúde. Os rins, que desempenham um papel fundamental na manutenção da homeostase volêmica, são especialmente vulneráveis a essas alterações.

O aquecimento global aumenta o risco de estresse térmico e desidratação, que são fatores que contribuem significativamente para injúria renal aguda e formação de cálculos renais^27,28^ e, em última instância, progressão para DRC^29^. Trabalhadores que exercem atividades ao ar livre, expostos ao calor extremo, estão particularmente em risco, sobretudo quando a hidratação é inadequada ou as condições de trabalho não estão suficientemente adaptadas, como ocorre na nefropatia mesoamericana, uma forma de DRC rapidamente progressiva identificada em trabalhadores agrícolas em climas (sub)tropicais^30^.

As mudanças climáticas também intensificam eventos climáticos extremos, como inundações, furacões e tufões. Esses eventos, combinados ao aumento das temperaturas, favorecem a disseminação de doenças tropicais, como malária e dengue, bem como de doenças transmitidas pela água, como leptospirose e diarreia infecciosa^31^, todas capazes de causar injúria renal aguda. As águas das enchentes também podem se tornar nefrotóxicas quando contaminadas por poluentes industriais ou naturais. Além disso, a exposição ao material particulado fino proveniente da indústria, dos transportes e de incêndios florestais têm sido associada à prevalência de DRC^32^.

Populações vulneráveis sofrem as consequências mais graves desses riscos, vivendo frequentemente em ambientes com proteção inadequada e acesso limitado a sistemas de climatização, áreas verdes e condições de trabalho seguras.

Em 2024, a 77ª Assembleia Mundial da Saúde adotou uma resolução histórica sobre Mudanças Climáticas e Saúde, reconhecendo a crise ambiental como uma grande ameaça ao bem-estar humano e reivindicando sistemas de saúde resilientes às mudanças climáticas e com baixas emissões de carbono. A Resolução instou os Estados-Membros a incorporarem a saúde às estratégias climáticas nacionais e endossou mecanismos como a Aliança para Ação Transformadora sobre Clima e Saúde, a fim de apoiar a implementação^33^.

Dando continuidade a esses avanços, a 78ª Resolução enfatizou especificamente a necessidade de uma ação global coordenada para combater as ameaças ambientais à saúde em geral e à saúde renal. Em conjunto, essas Resoluções estabelecem um duplo mandato imperativo: posicionar a saúde renal na interseção entre o controle das doenças não transmissíveis e a gestão ambiental responsável, e orientar os Estados-Membros no sentido de respostas de saúde mais integradas, equitativas e sustentáveis.

No entanto, o enfrentamento dos riscos ambientais deve caminhar lado a lado com a redução da pegada ecológica decorrente dos próprios cuidados renais (ver abaixo).

## CUIDADOS ECOLOGICAMENTE SUSTENTÁVEIS NA INSUFICIÊNCIA RENAL: RUMO A UMA NEFROLOGIA MAIS VERDE

A abordagem ideal para prevenir o impacto negativo das intervenções consiste em adotar medidas que garantam que elas deixem de ser necessárias. Nesse sentido, a prevenção de doenças renais contribui para a sustentabilidade do planeta ao retardar ou evitar a diálise e reduzir o uso de medicamentos, cuja fabricação também gera uma pegada ambiental. No entanto, se os medicamentos retardarem a progressão da DRC e a necessidade de terapia renal substitutiva (TSR), isso poderá compensar a pegada de carbono relacionada à produção desses medicamentos. Uma análise secundária de dados do estudo CREDENCE, controlado por placebo, que avaliou o impacto da canagliflozina nos desfechos, identificou uma redução de 20% a 25% nas emissões de gases de efeito estufa ao longo de 2,6 anos de acompanhamento em pacientes com diabetes tipo 2 que ainda não estavam em TRS^34,35^.

## TRANSPLANTE: PROMOVENDO O ACESSO E A SUSTENTABILIDADE

Entre as modalidades de TRS, o transplante oferece os melhores desfechos – custos sociais mais baixos, maior sobrevida, melhor qualidade de vida e impacto ambiental significativamente menor em comparação com a diálise^36,37^.

No entanto, o acesso ao transplante renal varia amplamente entre os países e dentro deles, sendo influenciado pela infraestrutura de saúde, pelo nível socioeconômico e pela localização geográfica (áreas rurais vs. urbanas)^4^. As práticas de doação também diferem: alguns países dependem fortemente de doadores vivos, enquanto outros se concentram principalmente na doação por doadores falecidos. Em muitas regiões, a doação após morte cardíaca ou o transplante preemptivo ainda são subutilizados, apesar dos desfechos favoráveis^38,39^. Além disso, potenciais doadores e receptores são frequentemente desqualificados com base em critérios arbitrários ou em preconceitos, o que exclui grupos sociais específicos, faixas etárias, mulheres, indivíduos com comorbidades não essenciais e doadores limítrofes. A exclusão de doadores limítrofes também reduz o número de doadores disponíveis, apesar das evidências de que os rins provenientes desses doadores são seguros, desde que haja avaliação cuidadosa, acompanhamento de longo prazo e tratamento dos fatores de risco^40^. Em contextos com poucos recursos, os programas de transplante renal costumam ser subdesenvolvidos ou inexistentes, o que agrava ainda mais as desigualdades em saúde e aumenta o ônus econômico.

A redução das disparidades no transplante requer diretrizes claras e endossadas globalmente sobre planejamento de programas, marcos éticos e legais para combater a doação remunerada, além de apoio técnico e institucional por parte de países ou unidades com programas de transplante avançados. Os esforços também devem incluir programas de educação pública para ampliar o número de doadores e abordar questões culturais, religiosas e sociais.

## DIÁLISE SUSTENTÁVEL: REDUZINDO O IMPACTO AMBIENTAL E MELHORANDO O ACESSO

A maioria das pessoas em TRS recebe tratamento dialítico, apesar das muitas desvantagens mencionadas anteriormente^37,41^. Nos últimos anos, o impacto ambiental da diálise tem se tornado uma preocupação adicional.

O setor de saúde contribui significativamente para a degradação ambiental^42^. A diálise é um dos principais fatores contribuintes devido ao seu consumo repetitivo e prolongado de água e energia, às emissões de gases de efeito estufa e à geração de resíduos plásticos^6^. Embora as atividades terapêuticas sejam parte direta desse processo, aproximadamente 70% das emissões de gases de efeito estufa relacionadas aos cuidados de saúde provêm da cadeia de suprimentos, em grande parte associadas à fabricação, ao transporte e ao tratamento de resíduos^6,43,44^. Além disso, volumes substanciais de dialisato utilizado e de rejeito de osmose reversa são geralmente descartados pelo sistema de esgoto, o que constitui um problema específico em regiões áridas ou durante períodos de seca^6,45^.

São necessárias medidas urgentes: investimento em tecnologias de diálise sustentável que priorizem a segurança do paciente, reduzindo a toxicidade decorrente de microplásticos e eluatos e, ao mesmo tempo, melhorando a experiência do tratamento ao reduzir fatores estressantes, como o ruído excessivo dos equipamentos; revisão crítica dos procedimentos clínicos para reduzir a pegada ambiental sem comprometer a qualidade do tratamento; e maior transparência sobre os impactos da fabricação e do transporte e como eles são abordados (Figura 2)^6^. Essa responsabilidade requer a colaboração entre a indústria, médicos, pacientes, enfermeiros, engenheiros, químicos e cientistas ambientais.

As terapias domiciliares — diálise peritoneal e a maioria dos esquemas de hemodiálise domiciliar, exceto diálise diária prolongada — oferecem vantagens ambientais em comparação com a hemodiálise em centro especializado, incluindo menor necessidade de transporte de pacientes e profissionais, menor consumo de energia para controle da temperatura ambiente, menor consumo de eletricidade pelas estações de osmose reversa e, no caso dos sistemas compactos de hemodiálise e da diálise peritoneal, menor consumo de água. A diálise peritoneal tem uma pegada ambiental menor do que a hemodiálise em centro^43,46,47^, embora o transporte de seus insumos, mais volumosos, ainda possa gerar emissões substanciais, especialmente quando a distância do transporte é longa^46^. Sugere-se também que a maioria dos esquemas de hemodiálise domiciliar apresente um benefício em comparação com a hemodiálise em centro, exceto no caso da hemodiálise prolongada de 6 × 8 horas (fluxo de dialisato de 300 mL/min)^47^. A diálise incremental pode reduzir ainda mais o impacto ambiental, juntamente com a otimização do fluxo de dialisato^48^, desde que seja clinicamente adequada e que se obtenha o consentimento informado.

A diálise peritoneal e os sistemas compactos de hemodiálise já existentes podem também contribuir para ampliar o acesso à diálise em contextos com poucos recursos e durante situações de crise. Além disso, diversos sistemas que regeneram o dialisato por meio de sorventes estão atualmente em desenvolvimento ou em fase de testes clínicos preliminares, mas ainda não disponíveis para uso clínico em larga escala^49^. No entanto, para garantir sua disponibilidade em países emergentes, é necessário superar barreiras como impostos de importação ou custos de transporte (p. ex., por meio da fabricação local).

## TRATAMENTO CONSERVADOR ABRANGENTE: INDISPENSÁVEL, MAS FREQUENTEMENTE ESQUECIDO

O tratamento conservador abrangente ou paliativo constitui a terceira opção para o tratamento da insuficiência renal, juntamente com o transplante e a diálise. Essa abordagem concentra-se em maximizar a qualidade de vida por meio de suporte médico completo, sem recorrer à TRS^50^.

O tratamento conservador é particularmente adequado para pessoas frágeis e idosas, muitas das quais apresentam um rápido declínio do estado funcional e aumento da mortalidade no primeiro ano após o início da diálise^51^. Pessoas com DRC nos estágios 3 a 5 preferiram o cuidado conservador caso a diálise implicasse um aumento no número de consultas hospitalares ou restringisse sua capacidade de viajar^52^. Apesar disso, o tratamento conservador continua frequentemente subutilizado^50,53^.

Iniciativas educacionais voltadas tanto para pessoas com doença renal quanto para profissionais de saúde devem aumentar a conscientização e a compreensão sobre os cuidados conservadores. A ampla promoção dessa abordagem pode ajudar a garantir que as pessoas recebam tratamentos alinhados aos seus valores, circunstâncias e desejos, ao mesmo tempo em que reduz encargos médicos, econômicos e ambientais desnecessários.

## EMPODERAMENTO DO PACIENTE: O PROBLEMA ÓBVIO QUE SE EVITA ABORDAR

Muitas pessoas relatam que as informações fornecidas antes do início da TRS são de qualidade mediana a insatisfatória, particularmente em países com produto interno bruto mais baixo^54^.

A tomada de decisão paternalista permanece comum, negligenciando oportunidades para o planejamento precoce da modalidade de tratamento, a criação de acesso vascular e o início eletivo da TRS. Em contrapartida, a tomada de decisão compartilhada envolve ativamente os pacientes nas escolhas terapêuticas, aumentando a satisfação, a qualidade de vida, os desfechos e as taxas de transplante e de diálise domiciliar, além de reduzir a dependência da hemodiálise em centro especializado^54,55^.

As estruturas hospitalares atuais ainda são pouco acolhedoras para os pacientes, de modo que mesmo aqueles que se manifestam com veemência são pouco ouvidos. Unidades ambulatoriais menores, de fácil acesso e independentes dos hospitais, maior envolvimento dos enfermeiros nas consultas e grupos de discussão com participação ativa dos pacientes são possíveis opções inovadoras, juntamente com diversas iniciativas educativas e informativas detalhadas ao longo deste texto. O apoio de organizações de pacientes poderia contribuir para o avanço dessas iniciativas.

O cuidado pré-diálise eficaz, a previsão precisa de riscos e o planejamento estratégico das modalidades de tratamento são cruciais para aprimorar os desfechos e reduzir os custos com assistência médica. No entanto, poderiam ser otimizados, especialmente em áreas com recursos limitados, por meio da prevenção de encaminhamento tardio, fragmentação do atendimento, educação inadequada do paciente, baixa adesão ao tratamento e comunicação limitada.

O envolvimento dos pacientes também é fundamental na concepção de pesquisas, na interpretação de dados e no desenvolvimento de registros, contribuindo para direcionar os esforços a questões que realmente lhes são importantes. Os pacientes também devem ser incentivados a participar de programas de capacitação que promovam a autoconfiança e o desenvolvimento de habilidades de comunicação, capacitando-os a se tornarem parceiros ativos no tratamento e a moldar o futuro da saúde renal^56^.

## CUIDADOS RENAIS EM SITUAÇÕES DE CRISE: ADAPTAÇÃO A UM MUNDO EM TRANSIÇÃO

O número de pessoas afetadas por situações de crise vem aumentando consistentemente, impulsionado pelo crescimento populacional, pelas mudanças climáticas e pela crescente instabilidade geopolítica. Essas crises abrangem desde desastres naturais (terremotos, inundações, fenômenos climáticos extremos e pandemias) até emergências provocadas pela ação humana, particularmente conflitos armados^57^.

Pessoas com doenças renais são particularmente vulneráveis, uma vez que dependem de medicamentos especializados e de tratamentos que requerem muitos recursos, como a diálise. Esses tratamentos exigem equipe qualificada, água potável, energia elétrica, equipamentos em funcionamento, transporte para o abastecimento de insumos e infraestrutura confiável — recursos que geralmente estão comprometidos em situações de crise^57^.

A perda das sessões de diálise pode levar rapidamente a complicações potencialmente fatais, como a hipercalemia, que pode exigir evacuação urgente^58^. A interrupção das terapias medicamentosas pode acelerar a progressão da DRC ou causar rejeição do enxerto renal. Apesar desses riscos, pessoas com doenças renais raramente são consideradas nos planos oficiais de contingência.

À medida que as crises se tornam mais frequentes e graves, a saúde renal deve ser integrada aos planos de contingência e aos protocolos de emergência. As autoridades devem ser sensibilizadas para as necessidades específicas da comunidade nefrológica por meio de ações de defesa firmes e coordenadas. Caso os cuidados renais não estejam contemplados nos planos gerais de contingência, comunidades nefrológicas e nefrologistas devem elaborar seus próprios planos. Ao mesmo tempo, há uma necessidade urgente de estratégias de tratamento que sejam menos dependentes de recursos.

## CHAMADO À AÇÃO: UMA RESPONSABILIDADE COLETIVA E GLOBAL

O modelo tradicional de cuidados renais, centrado na hemodiálise em centros especializados, já não é sustentável — nem do ponto de vista ambiental, nem econômico, nem ético (Figura 1). Pessoas que convivem com DRC precisam urgentemente de melhor qualidade de vida e de melhores cuidados. A transformação requer uma abordagem holística (Quadro 1), na qual todas as partes interessadas considerem cada uma das áreas-chave discutidas anteriormente e as adaptem às situações, à infraestrutura e aos recursos locais.

O DMR 2026 reforça essa necessidade, lançando um apelo à ação global:

- A população deve ser orientada a questionar os profissionais de saúde sobre seus parâmetros renais e função renal.
- Os trabalhadores, particularmente aqueles que exercem atividades ao ar livre nos setores agrícola e de construção civil, devem ser conscientizados sobre os riscos ocupacionais decorrentes do calor e da poluição ambiental, bem como sobre as estratégias para mitigar esses riscos, a fim de preservar a saúde renal.
- As pessoas com doença renal e suas comunidades devem ter autonomia para desempenhar um papel ativo na definição de trajetórias de cuidado que atendam às suas necessidades e defendam seus interesses.
- Os profissionais de saúde devem mudar o foco para um cuidado integrado, preventivo e baseado na comunidade, que promova o bem-estar e a responsabilidade ambiental, com ênfase nos estágios iniciais da doença e em abordagens fora do ambiente hospitalar.
- Pesquisadores e indústria devem desafiar o *status quo* e priorizar a inovação, buscando equidade, flexibilidade, acolhimento ao paciente e sustentabilidade.
- Os formuladores de políticas e administradores precisam reconhecer a síndrome cardio-renal-metabólica como uma ameaça crítica à saúde pública, o que exige uma reformulação do sistema e a promoção de cuidados renais sustentáveis por meio de iniciativas de financiamento e reembolso.
- As organizações de defesa dos pacientes devem ser reconhecidas como parceiras essenciais na concepção, implementação e avaliação de iniciativas de políticas públicas, a fim de garantir que o sistema seja estruturado em torno das necessidades específicas dos pacientes. No entanto, a mobilização ativa é uma responsabilidade de toda a comunidade envolvida no cuidado renal.
- As parcerias globais na área da saúde devem articular esses esforços, promovendo, em todos os níveis, cuidados renais e uma saúde global coesos e preparados para os desafios futuros.

A falta generalizada de conscientização — entre população, formuladores de políticas e até mesmo profissionais de saúde — contribui para as desigualdades persistentes em termos socioeconômicos, de gênero (identidade social ou cultural, e não biológica), étnicos, nacionais e regionais. Para combater essa situação, são necessárias ações educativas robustas que destaquem as ameaças à saúde pública e o ônus global impostos pelos danos renais e pelas doenças renais.

Dadas as tendências demográficas e geopolíticas, o rastreamento, a detecção precoce e a prevenção, quando adequados e bem planejados, constituem os primeiros e mais importantes passos para alcançar as metas de sustentabilidade. Essas medidas reduzem as complicações, a progressão para a doença renal avançada e a necessidade de TRS. Em última análise, a prevenção também beneficiará a saúde global ao mitigar o curso de doenças que frequentemente causam ou agravam a DRC e/ou são aceleradas por ela, reduzindo significativamente os problemas individuais e sociais que surgem mesmo em sistemas de seguridade social robustos. Na ausência de cobertura universal, isso trará benefícios ainda maiores, ao evitar desfechos fatais precoces e consequências financeiras devastadoras para muitos.

## CONCLUSÃO: ALINHANDO A SAÚDE RENAL ÀS AGENDAS GLOBAIS DE SAÚDE E SUSTENTABILIDADE

O reconhecimento das doenças renais como uma prioridade global pela Organização Mundial da Saúde na 78ª Assembleia Mundial da Saúde marca um momento decisivo^5^. Esse reconhecimento proporciona um mandato abrangente para ações urgentes e uma base sólida para a defesa de causas em âmbito nacional e internacional.

O tema do DMR 2026 impulsiona a integração da saúde e dos cuidados renais a agendas mais amplas de saúde e sustentabilidade. Essa transição para modelos centrados no paciente deve ser defendida por todas as partes interessadas que compartilham a responsabilidade pela concretização dessa abordagem nos setores da saúde, meio ambiente, trabalho e políticas públicas, por meio de parcerias, movimentos e coalizões alinhados, com grupos que compartilham interesses paralelos em diversas especialidades médicas.

Esta publicação e o DMR 2026 lançam um apelo urgente e conjunto para que se priorize a saúde renal como pilar de um futuro mais saudável, justo e sustentável. As decisões tomadas agora determinarão a vida de milhões de pessoas nas próximas décadas.

## Disponibilidade de dados

Nenhum dado novo foi gerado ou analisado neste estudo.
